# Cervical, Thoracic, and Lumbar Spine Epidural Abscess: Case Report and Literature Review

**DOI:** 10.1155/2020/8834589

**Published:** 2020-10-07

**Authors:** Bohdan Baralo, Mrunal Kulkarni, Rithikaa Ellangovan, Robert Selko, Ajinkya Kulkarni, Shambo Guha Roy, Mark Gilbert

**Affiliations:** ^1^Internal Medicine, Mercy Catholic Medical Center, Darby, PA, USA; ^2^Radiology, Mercy Catholic Medical Center, Darby, PA, USA; ^3^Infectious Diseases, Mercy Catholic Medical Center, Darby, PA, USA

## Abstract

We report a case of a spinal epidural abscess (SEA) in a patient without significant risk factors. The patient was treated in an outpatient setting for one week for worsening back pain and subsequently admitted to the hospital for the treatment of sepsis and suspected SEA. An MRI obtained on admission showed an epidural abscess extending from the lower cervical to the upper lumbar region and accompanying paraspinal cervical and psoas abscesses. The patient was successfully treated with antibiotics based on the sensitivity of the surgical cultures received from a needle aspiration of the abscess. SEA has a low incidence; however, the number of cases is consistently rising over the last two decades. The outcome of SEA treatment is related to the duration of the process prior to intuition of the treatment. Patients with no neurological symptoms, or with symptoms lasting less than 36 h, have the best recovery rate. As the typical symptoms of SEA are seen in only 13% of cases, physicians should have a low threshold to order MRI in patients with back pain that is new or changed from the baseline. With the help of CT-guided aspiration for culture analysis, patients can be successfully treated conservatively using antibiotics in cases where neurological signs are absent.

## 1. Introduction

Spinal epidural abscess (SEA), although rare [1], has exhibited a rising incidence in recent years [2]. This disease can often mimic more benign musculoskeletal alignments and delay the timing of diagnosis [3]. The outcome of the disease is much dependent on the severity and duration of the symptoms before intervention, and successful resolution depends upon early recognition [2]. In our report, we present a case of an unusually large posterior SEA presenting with atypical symptoms and successfully treated with conservative treatment only.

## 2. Case Description

A 57-year-old woman was admitted to the hospital with one week of worsening, nontraumatic back pain. The patient reported a history of chronic back pain secondary to her sedentary occupation. The pain had significantly worsened from baseline approximately seven days prior to admission. Before arrival, the patient was prescribed a lidocaine patch without significant relief. The pain did not radiate and was appreciated at the level of the thoracic spine. The pain intensity, 10/10, was aggravated by motion and relieved with rest, and to some degree, with hydromorphone. The patient's past medical history was significant for COPD, hyperlipidemia, depression, bipolar disorder, anxiety, GERD, irritable bowel syndrome, and distant history of the intravenous drug abuse with heroin in her youth. The patient's home medications included: albuterol inhaler, BREO ellipta, cyclobenzaprine, acetaminophen, ibuprofen, aspirin, docusate, ondansetron, ranitidine, and a lidocaine patch. The review of systems was otherwise negative.

On physical examination, no skin changes were detected over the patient's back. The spine was tender to palpation in the paraspinal area and tender to percussion at the midline. The neurologic examination performed at the bedside did not show any gross or focal neurologic signs. The lungs were clear to auscultation bilaterally. Heart tones were clear, without rubs, murmurs, or gallops. The rest of the physical exam was unremarkable. No skin tracks or other signs of intravenous drug abuse were detected during the physical exam.

The Emergency Department vital signs observed an oral temperature of 98.3°F, a blood pressure of 147/100 mmHg, a pulse of 96/min, and respirations of 20/min. O_2_ saturation was 98% on room air. During her time in the emergency department, blood pressure was normalized.

The laboratory assay revealed aWBC count of 26.100 leukocytes/*μ*l with 78% neutrophils, an elevated lactate of 2.4 mmol/l, a sodium level of 118 mEq/l, a chloride level of 78 mEq/l, CRP >320 mg/l, an ESR 75 mm/h, and a creatinine level of 1.9 mg/dl which was three times more than her baseline. Urinalysis was normal. Chest X-ray done on admission showed hypoinflation with areas of linear airspace disease representing atelectasis and a small left pleural effusion.

The patient was admitted to the hospital with a diagnosis of sepsis with a concern for spinal infection, hyponatremia, and acute kidney injury. She was prescribed broad-spectrum antibiotics (ceftriaxone, vancomycin, and metronidazole) and normal saline. Infectious Diseases and Neurosurgery services were consulted.

**Figure 1:**
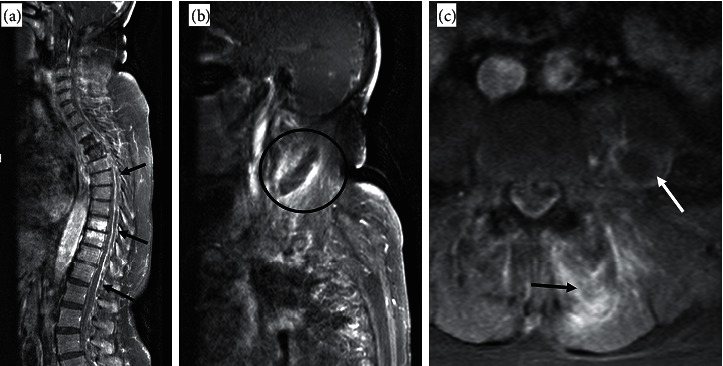
(a) Limited T1 postcontrast whole spine image showing enhancing posterior epidural collection (black arrows) from the level of C3 (not seen in the image) till L1. (b) Sagittal T1 postcontrast views at the cervical level, showing enhancing abscess around the right sternocleidomastoid muscle (black circle). (c) Axial T1 postcontrast images at lumbar levels showing enhancement of the left paraspinal muscles (black arrow) and additional abscess within the left psoas muscle (white arrow).

An MRI was performed revealing (1) a large posterior SEA extending from the lower cervical spine into the upper lumbar spine causing moderate spinal canal narrowing with mild cord compression in the lower thoracic spine without abnormal signal to suggest cord edema or ischemia, (2) extensive cellulitis and myositis involving the paraspinal musculature of the cervical and lower lumbar spine, (3) small left paraspinal abscesses of the lumbar region, and (4) large right cervical paraspinal abscesses and a left psoas muscle myositis with a 2 cm left psoas abscess (Figure 1).

Neurosurgery service deferred an open surgical procedure and recommended a guided aspiration of the abscess for diagnosis and culture. Antibiotics were adjusted to cefepime and vancomycin. A lumbar puncture was not deemed necessary.

The ultrasound-guided percutaneous aspiration of the right neck paravertebral abscess yielded only 3 cc of highly viscous purulent fluid. The patient was transferred to the intensive care unit for close postprocedure monitoring. A 2D echocardiogram performed on the day of admission did not show any definite vegetation on the cardiac valves. Cultures obtained from the cervical abscesses grew pan-sensitive *Staphylococcus aureus*. The patient's antibiotic therapy was adjusted to nafcillin.

The patient remained stable after procedure with no exacerbation of her neurologic status and a diminishment in the intensity of her back pain.

As her clinical status improved, the patient was discharged to a skilled nursing facility to complete a 12-week course of nafcillin.

Six weeks after initiation of the antibiotics course, the patient's leukocyte count decreased to 10.9, and the basic metabolic panel were within normal limits. A repeat MRI performed eight weeks after initiation of antibiotic therapy showed resolution of previously seen paraspinal abscesses and phlegmonous changes. A repeat physical exam did not show any focal neurologic changes, and the patient's back pain was back to baseline.

## 3. Discussion

In 1975, the incidence of a spinal epidural abscess was estimated by Baker et al. to be 0.2–1.2 cases per 10,000 hospital admission per year [4]. Recent data from a 2017 reported by Vakili and Crum-Cianflone estimated an increase of SEA incidence to 5.1 cases per 10,000 entries [1]. The incidence rate varies depending on location [5] with the variability secondary to increased utilization of the MRI, increased availability of imaging technology, and increased number of predisposing conditions [1].

The early signs and symptoms of SEA are vague. The classic symptoms including back pain, fever, and neurologic deficits occur in only 13% of cases at the time of diagnosis [6]. The outcome of SEA depends upon the severity and duration of neurologic deficits before both antimicrobial and surgical intervention [2]. The absence of paralysis, or its presence for <36 h, is associated with better survival and return of function [6].

The proper interview of the patient should evaluate for risk factors associated with the disease, including:

1. Compromised immunity by diabetes mellitus, steroid or other immunosuppressive therapy, malignancy, pregnancy, HIV infection, alcoholism, and cirrhosis.
2. Disruption of the spinal column by degenerative disease, trauma, surgery, or instrumentation, including discography, chemonucleosis, and central neuraxial block, provide a direct portal for organisms. Even temporally distant blunt trauma is a risk factor.
3. Sources of infection include respiratory, urinary, and minor soft tissue infections and may all act as primary sources of hematogenous spread. Intravenous drug abusers and patients with indwelling vascular catheters are constantly at risk.

MRI imaging with gadolinium is the gold standard of imaging for suspected SEA.

Most SEAs are located posterolateral of the thoracic and lumbar region where the epidural space is the largest. Because there is no barrier to the axial spread of infection, often 3 to 5 segments are involved [3]. However, the abscess can extend beyond a particular spinal region. Barner et al. reported the case with the involvement of 7 segments [7]. Most cases of the anterior SEA are associated with vertebral osteomyelitis and located near the affected vertebral body [2].

The exact mechanism of spinal cord damage is unclear as the degree of neurologic dysfunction is often out of proportion of radiologic changes. This true in both ways: tetraplegia with a patient's subarachnoid space was described, as well as a complete obliteration of subarachnoid space with completely intact neurologic function [6].

The laboratory test results are nonspecific and usually look the same as sepsis due to other sources (high WBS, ESR, and CRP). Microbiology testing with sensitivity is necessary to guide antibacterial treatment. A large variety of pathogens have been found as causative agents for SEA including mycobacteria, fungi, and parasites. Bacterial pathogens include Gram-negative rods (*E. coli*, *P. aeruginosa*, and *Klebsiella* spp.) and *Streptococcus* spp. (*Viridens* group, *S. agalactiae*, and *S. pneumoniae*) with *Staphylococcus aureus* by far the most frequent [2, 6].

Lumbar puncture plays a less critical role in diagnosing SEA and should not be performed routinely. Neither Gram staining (generally negative) nor cultures of CSF (growth in 6–28%) reveal results with acceptable sensitivity. The cell count is usually elevated but varies widely [2].

The treatment of epidural abscesses is a complex decision that often requires a multidisciplinary approach. Antibiotics alone can be used under close supervision in patients diagnosed with SEA and whose neurologic findings are absent. The duration of antibiotic therapy is usually 4–6 weeks with extension to 8–12 weeks when there is concomitant osteomyelitis of the vertebral bodies [1, 2, 8]. In most cases, nonsurgical treatment failure is apparent during the first 48–72 h after the onset of therapy, though failure may occur at later stages [2]. Historically, surgical treatment included laminectomy with decompression and drainage of the abscess [3]. A combination of surgery with antibiotics remains the most often recommended treatment [1, 2, 5].

CT-guided percutaneous drainage is also a rapidly evolving treatment modality for carefully selected patients (no neurological deficit, high surgery risk, and patients who did not respond to the initial antimicrobial treatment) [1, 2]. An increasing number of case reports regarding the successful utilization of CT-guided aspiration have been published. Ross et al. reported two cases with intact neurologic symptoms (one with concomitant osteomyelitis) that were managed with CT-guided aspiration and antibiotics with good response [9]. Ran et al. reported CT-guided treatment of a patient with a large SEA extending from L2 to S2 who failed antibiotic therapy. Surgery was performed after the patient began to develop sensory deficits. CT-guided aspiration from 3 foci and washout of the epidural space with gentamycin was performed. The antibacterial treatment was adjusted according to sensitivity of the culture obtained during the procedure. Neurologic signs resolved, and a 2-month follow-up MRI showed complete resolution of the abscess [10].

## 4. Conclusion

Spinal epidural abscess, a rare diagnosis on admission, with a classic triad (fever, back pain, and neurologic deficit) may present without typical risk factors as happened in our case. The neurologic changes can be variable and range from absent focal deficits to tetraplegia without significant changes in imaging. The diagnosis of SEA should be considered in patients with significant back pain in conjunction with SIRS criteria, especially when no other obvious source of infection can be identified. The threshold for ordering MRI should be low and includes cervical, thoracic, and lumbar spine segments in order not to miss the potential source.

The antibiotics are the mainstay of treatment with the option of direct surgical treatment depending on the severity of neurologic symptoms. The extent of abscess involvement is not an indication for surgery when there is no neurologic deficit and proper response to the initial antibacterial therapy observed. Constant vigil of the patient's neurologic status, repeat MRI, and identification of pathogens with sensitivity results are essential for obtaining and confirming resolution of infection.

## Data Availability

The data used to support the findings of this case report are restricted in order to protect the patient's privacy.
